# How Do Medical Students Respond to Emotional Cues and Concerns Expressed by Simulated Patients during OSCE Consultations? – A Multilevel Study

**DOI:** 10.1371/journal.pone.0079166

**Published:** 2013-10-23

**Authors:** Yuefang Zhou, Alex Collinson, Anita Laidlaw, Gerry Humphris

**Affiliations:** School of Medicine, University of St Andrews, St Andrews, United Kingdom; George Mason University, United States of America

## Abstract

**Objectives:**

How medical students handle negative emotions expressed by simulated patients during Objective Structured Clinical Examinations (OSCE) has not been fully investigated. We aim to explore (i) whether medical students respond differently to different types of patients’ emotional cues; and (2) possible effects of patients’ progressive disclosure of emotional cues on students’ responses.

**Methods:**

Forty OSCE consultations were video recorded and coded for patients’ expressions of emotional distress and students’ responses using a validated behavioural coding scheme (the Verona Coding Definitions of Emotional Sequence). Logistic multilevel regression was adopted to model the probability of the occurrence of student *reduce* *space* response behaviour as a function of the number of patients’ expressions of emotional cues.

**Results:**

We found that medical students offered responses that differed to emotional cue types expressed by simulated patients. Students appeared to *provide* *space* to emotional cues when expressed in vague and unspecific words and *reduce* *space* to cues emphasizing physiological or cognitive correlates. We also found that medical students were less likely to explore patients’ emotional distress nearer the end of the consultation and when the duration of a patient speech turn got larger. Cumulative frequency of patients’ emotional cues also predicted students’ *reduce* *space* behaviour.

**Practical Implications:**

Understanding how medical students manage negative emotions has significant implications for training programme development focusing on emotion recognition skills and patient-centred communication approach. In addition, the statistical approaches adopted by this study will encourage researchers in healthcare communication to search for appropriate analytical techniques to test theoretical propositions.

## Introduction

An empathic response to patient’s emotional needs is one of the key features of patient-centred care [1–4]. This approach, marked by a provider’s ability to recognize and manage their patients’ emotional cues, is associated with a variety of positive patient care outcomes [5–9]. There are increasing pressures to improve patient care outcomes in public and private health care organisations. Providers are required to see more patients within the time and resource they have available [10]. Hence it is important to research the conditions that promote positive responses to patients’ troubling emotions. 

Studying the doctor’s response to patient’s concerns and emotional cues has remained a focus in healthcare communication research for more than a decade [11–18]. The methodologies adopted by many studies, however, have been limited to sequential analysis of purely conversational variables at a single level. The reported findings are therefore often de-contextualized. Multilevel sequential analysis has recently emerged as an encouraging development to study the communication process [19–22]. One pioneering study in this area was conducted by Del Piccolo and her colleagues [20], who used multilevel binary logistic regression to demonstrate how cue expressions were associated with factors relating to patient, doctor, the setting, as well as communication sequences. The study conducted by Romondini et al. [19] is among the few that has explored doctors’ responses to patient cues and concerns in a multilevel fashion. They grouped responses into four categories using the Verona Psychiatric Interview Classification System (VR-PICS) [19,23] and modelled psychiatrists’ immediate response to simulated patients’ speech type using multinomial regression. It was found that patients’ expressions of concern comprehensively increased the probability of passive listening. However two specific types of expressions of concern (patient statements of feelings and expressions of opinions regarding problematic psychological issues) elicited active listening. Their findings have encouraged further investigation about how different types of concern expression might affect doctor responses. Employing a similar approach, Del Piccolo et al. [21] categorised responses into three groups using the Verona Coding Definitions of Emotional Sequence (VR-CoDES) [24,25]: (i) provide space in a non-explicit way, providing space with explicit reference to either (ii) the affective or (iii) the factual content. Using multinomial regression, they modelled the immediate responses of psychiatrists to cues and concerns of the patient considering patient, psychiatrist and consultation variables. Researching impacts of provider response to patient emotionally charged expressions on patient outcomes has received increasing attention in recent years [26,27], no studies, however, have yet researched the communication influences on health outcomes adopting a multilevel approach. 

In light of these current developments on multilevel studies about emotions in healthcare communication, no exploration has reported how medical students respond to simulated patient’s negative emotions in their Objective Structured Clinical Examinations (OSCE). In particular, it is of interest to know how different cue types may elicit different responses in an OSCE context. There are important implications for clinical training and practice which would stem from such an investigation. For example, it would be important to know if students, relatively early in their training, varied in a systematic way to respond to cues and concerns elicited by simulated patients. It may be possible to manipulate the provision of cues and concerns in simulated patients and observe student responses. This would add to our knowledge of clinician recognition and response to emotional elements of communication and hence improve our training interventions. 

Since its development in 2001, the VR-CoDES has been successfully applied to a variety of contexts ranging from psychiatric interviews [21], paediatric consultations [28], to the dental context [29] and email communications [30]. It is the first time that the authors are aware that the VR-CoDES has been applied to clinical communication stations within an OSCE in such a manner focusing simultaneously on the detailed consultation processes and the personal qualities of the actors involved. We are, therefore, interested in testing the applicability of the VR-CoDES in an OSCE context. 

To date the majority of studies investigating the relationship between patient cue expression and provider response have adopted a ‘lag 1 sequential analysis’ approach [13,15,16]. While it is important to understand communication influences between adjacent utterances, the limitation of this approach is that the longer-term speech influences remained unexplored. The extension of these effects across the consultation might be potentially very important [22]. Hence in our study we also attempted to examine ‘longer-term and more strategic communication processes’ [22], focusing on cumulative effects of patient cue disclosure on medical student behaviour. This investigation requires a closer focus on multilevel sequential analysis of lag-independent relationships. In the medically unexplained symptoms (MUS) literature, this approach has been described as resembling an ‘attrition’ process and it has been used to explain how patient strategic presentation of symptoms affects doctors’ choice of treatment recommendations [31]. Salmon et al [31] found that, in a multilevel logistic regression model, the log odds of a GP responding somatically was a function of the total number of times that the patient complained about the symptoms prior to the point when a somatic intervention was offered. This model of communication may be readily applied to other settings more generally, such as the Irritable Bowel Syndrome (IBS). Such a patient scenario was designed for our study and has some parallels with a MUS patient presentation. While investigating medical students’ immediate responses to different cue types, we also aimed to explore how simulated patients progressively extend disclosure of their emotional cues and the effect this has on student responses.

Given the background described above, we attempted to seek answers to the following research questions:

1. Is the VR-CoDES applicable to an OSCE consultation context?
2. Do medical students respond differently (*provide* *space* vs *reduce* *space*) to different cue types defined by the VR-CoDES?
3. Do cumulative frequencies of specific cue types have an effect on the occurrence of *reduce* *space* response?
4. Is response type (*provide* *space* vs *reduce* *space*) related to the OSCE outcome, as rated by an expert examiner and the patient?

It is hypothesized that different cue types expressed by simulated patients stimulate different responses. The progressive disclosure of certain type of cues by patients may also influence the students’ choice of response. 

## Methods

### Participants

Forty 2nd-year medical students (20 males) at the University of St Andrews (2009-2012) participated, 29 of whom performed consistently poorly in clinical communication in previous OSCEs based on examiner scores. They all received a 2 x 2 hour communication skills training sessions focusing on identifying weak areas and providing opportunity to practise simulated patient consultations with feedback. One week after the end of the training sessions, they sat a practice clinical communication OSCE station with one of two simulated patients (age around 40) using an Irritable Bowel Syndrome scenario. After the student completed the OSCE consultation, both the examiner and the patient rated the student’s performance. The examiner scored the student on various components which could be grouped under seven different themes: introduction (maximum score = 2), presenting problem (maximum score = 2), relevant clinical information and risk factors (maximum score = 10), response to patient concerns (maximum score = 2), global communication (maximum score = 12), closing (maximum score = 6) as well as potentially a maximum score of 2 for merit. Among these seven themes, response to patient concerns and global communication were most relevant to their responses to cues and concerns. For the current study the total score (maximum = 36) generated from the seven themes was used for data analysis (Mean = 25.25, *SD* = 3.59). The patient’s scores on whether the student listened and responded to his/her concerns (0 = did not respond, 1 = unsure, 2 = responded) were used for this study (Mean = 1.70, *SD* = 0.46). 

### Ethics statement

This study was conducted in accordance with ethical standards laid down in the 1964 Declaration of Helsinki and was approved by the institutional review board of the University of St Andrews Teaching and Research Ethics Committee (UTREC) on 23 March, 2009 (human subjects research protocol MD5316). All student and simulated patient participants gave written informed consent to have their OSCE sessions video recorded on the Medical School’s integrated digital storage system and for it to be used for research purposes. All consent procedures used in this study were approved by the UTREC. 

### Coding cues/concerns and responses

The *Verona Coding Definitions of Emotional Sequence* (VR-CoDES-CC and VR-CoDES-P) [24,25] was used to code simulated patient cues/concerns and student responses. According to the manual, cues are defined as ‘verbal or non-verbal hints, which suggests an underlying unpleasant emotion that lacks clarity.’ Concerns are defined as ‘clear and unambiguous expressions of an unpleasant current or recent emotion that is explicitly verbalised with or without a stated issue of importance’ [24]. Cues are then further distinguished into seven subcategories according to the way emotional talk is introduced by the patient and the content by which the emotion is expressed [21,24]. As we believe that provider response is likely to be influenced by cue types, typical examples are illustrated here to demonstrate how cues were coded according to their distinguished types (Table 1). 

**Table 1:** Definitions of cues and concerns and examples from the OSCE* consultations.

**Definitions**	**Typical examples from the OSCE consultations**
**CONCERN**: a clear and unambiguous expression of an unpleasant current or recent emotion where the emotion is explicitly verbalized with or without a stated issue of importance.	‘But I am worried about this episode.’ ‘I am also feeling quite anxious.’ ‘I’m quite frustrated by it.’
**CUE**: a verbal or non-verbal hint suggests an underlying unpleasant emotion and would need a clarification from the health provider. Instances included:	
**Cue A**: vague or unspecified words or phrases in which the patient uses to describe his/her emotions.	‘Well, I am very stressed at work at the moment.’ ‘I just don’t know and I’m bit fed up with it, to be honest.’ ‘Yes, exactly, so I’m kind of embarrassed about it as well.’
**Cue B**: verbal hints to hidden concerns (emphasizing, unusual words, unusual description of symptoms, profanities, exclamations, metaphors, ambiguous words, double negations, expressions of uncertainties and hope).	‘It’s actually causing me considerable discomfort now.’ ‘But it’s getting worse; there must be something they can do.’ ‘And I have a horrible bloated feeling.’
**Cue C**: words or phrases that emphasizes (verbally or non-verbally) physiological or cognitive correlates (regarding sleep, appetite, physical energy, excitement or motor slowing down, sexual desire, concentration) of unpleasant emotional states.	‘And also I can’t really have my mind on other things when I am teaching.’ ‘I don’t really notice any other effect other than making me very tired.’ ‘You know if you are in a lot of pain, you just can’t concentrate on anything.’
**Cue D**: neutral expressions that mention issues of potential emotional importance which stand out from the narrative background and refer to stressful life events and conditions.	‘I found it quite difficult at work and it’s just generally very disruptive.’ ‘It’s very difficult particularly at work, cause it causes quite a lot of discomfort.’ ‘She (the head teacher) tries to get everything changed around and just driving everyone insane.’
**Cue E**: a patient elicited repetition of a previous neutral expression (repetitions, reverberations or echo of neutral expression within a same turn are not included).	Turn 1. ‘I also would quite like to have it investigated and see what’s causing it long term or if this is just something I have to put up with.’ Turn 2. ‘You know and something really needs to be investigated.’
**Cue F**: non-verbal cues including clear expressions of negative or unpleasant emotions (crying) or hint to hidden emotions (sighing, silence after provider question, frowning etc.).	‘Well previously it was every couple of 2-3 months (big sigh, then silence).’ ‘As far as I can see (sigh).’
**Cue G**: a clear and unambiguous expression of an unpleasant emotion which occurred in the past (more than one month ago) or is without time frame.	Not occurred.

* OSCE = Objective Structured Clinical Examination

Provider responses were coded for both dimensions: whether the response *explicitly* or *not explicitly* refers to the cue/concern; and whether the response *provides space* or *reduces space* for further disclosure of the cue/concern. For the purpose of the present paper, only *provide vs reduce* space dimension is considered. 

The coding procedure is implemented through application of the VR-CoDES onto The Observer XT^®^ 8.0 [32], a system for collection, analysis and presentation of observational data. The Observer is a trademark of Noldus Information Technology. Apart from frequency of cues/concerns and responses, we have obtained additional information through The Observer system that is important to understand the cue/concern and response relationship. Additional data included duration of consultation, time location where cues/concerns occurred during the consultation and the duration of the patient speech turn where a cue/concern was expressed. Two trained researchers (AW as an overall coder and YZ for reliability checks) coded the 40 video tapes over a 10-month period between 2011 and 2012. Cohen’s Kappa [33], with 95% confidence interval estimates, was used to check both inter- and intra- coder reliability.

### Data analysis

A nested data structure of consultation was considered, where speech turn is nested within consultation, which was in turn nested within students. Our outcome variable was student response to cues/concerns, coded as either *provide space* or *reduce space* to each cue/concern observed. For the convenience of analysis, we used *reduce space* as our outcome variable. Explanatory variables at the speech turn level (Level 1) included: occurrence of a specific type of cue and concern (1 = yes, 0 = no), cumulative frequency of a certain type of cue and concern, duration of a patient speech turn where a cue/concern occurred, time location when a cue/concern was expressed relative to the beginning of the consultation (first utterance start time). Predictive variables at the student level (Level 2) included: student gender (1 = female, 0 = male), consultation duration, rating scores by an examiner and the simulated patient.

Preliminary analysis described the data, presenting the frequency for cues/concerns and responses. Chi-square tests were then performed to explore response frequency differences (*provide space* vs *reduce space*) to concerns and each type of cues. An adjusted residual analysis [34] was then followed to confirm where the difference between observed and expected frequencies was relevant for the next-stage exploration. Two types of logistic multilevel regression models were fitted for binary outcome variable *reduce space*, using maximum likelihood via adaptive Gaussian quadrature estimation methods in statistical package STATA/IC™ 10.0 for Windows. Model Type A used lag 1 sequence analysis to model the log odds of the immediate *reduce space* response to concerns and specific type of cues, to address the research question two. Model Type B adopted an ‘attrition’ model to help explain the probability of the *reduce space* response to cumulative frequency of concerns and specific type of cues, to answer research question three. All variables in both models were entered progressively according to the criteria established in the following 5-step procedure: (1) A null model with random intercept explored variance composition in each level. (2) All Level 1 and then all Level 2 variables were entered separately into the *xtmelogit* procedure to explore relative effects of each variable on the log odds of the outcome variable. Those variables with a significant effect at *p* < 0.05 level, as well as those with theoretical and/or practical significance, were considered to be retained for further analysis (3). All variables are initially modelled with fixed effects due to limited variability in Level 2 variables. In addition, the nonlinear effect of cue emission time was tested (4). Effect of gender was controlled for in the final model for practical considerations (5). All models were checked for improvement in comparison to the previous one when additional predictors were added or removed. All continuous Level 2 variables were entered grand mean centred. 

## Results

### Applicability of the VR-CoDES to OSCE consultations

Both inter- and intra-coder reliability was checked on separate randomly selected clips at different coding periods. Agreement was accepted for identification of cues and concerns as well as type of cues. If *provide space* code was applied by both coders, but in different dimension of *explicit* or *non-explicit*, it is regarded as disagreement. The main disagreement lay in the interpretation of *Cue A* and *concern* in this particular context. When about three quarters of coding (28 clips) was completed, an intra-coder reliability was calculated (*k* = 82%) indicating a consistent coding procedure for the main coder. The inter-coder reliability was checked twice when approaching the end of the coding and was improved on the second occasion. This was achieved through strict compliance with the coding definitions and detailed discussion between coders. It is also reassuring that an expert from the VR-CoDES centre in Verona confirmed over 80% of the codes identified by the main coder on three selected clips. The overall Cohen’s Kappa was considered satisfactory according to Altman’s criteria [35]. Table 2 summarizes the results of inter- and intra-coder reliability. 

**Table 2:** Results of inter- and intra-coder reliability (*n* = 40).

**Type**	**Occasion of check**	**Cohen’s *K* (95% CI)**	**Agreement (%)**
Inter-coder	1^st^ time: end of 30 clips	0.78 (0.59, 0.97)	81.0%
	2^nd^ time: end of 40 clips	0.83 (0.67, 0.98)	85.7%
Intra-coder	End of 28 clips	0.82 (0.80, 0.88)	85.2%

### Frequency of cues/concerns and responses

Table 3 presents the results on the frequency for cues/concerns and student immediate responses to cues and concerns. On average, the number of the cues/concerns identified per consultation was about nine. Regarding the frequency rank, *Cue B* was most frequently observed, followed by *Cue A* and *Cue D* with same frequency. *Concern* was the third most frequently observed, whereas *Cue C*, *Cue E* and *Cue F* were rarely observed. No instances of *Cue G* were identified. Regarding responses, the chi-square tests indicated that students were more likely to *provide space* to *Cue A* and *reduce space* to *Cue C*, which was confirmed by the adjusted residual analyses (Adj. Res. = -2.97 for *Cue A*; Adj. Res. = -3.12 for *Cue C*). Among the 40 consultations, *Cue A* was observed in 36 of them (Mean = 1.55, *SD* = 0.96, *Min* = 0, Max = 4); whereas *Cue C* occurred in only 17 consultations (Mean = 0.45, *SD* = 0.55, *Min* = 0, Max = 2). 

**Table 3:** Frequency of cues of concerns and reponses.

**Cue type**		**Response**		**Cue/Concern per consultation**		**Chi-square**	
	**providing space**	**reducing space**	**total**	(***n*=40**)	**value**	***df***	***p***
**Concern**	38(64.41%)	21(35.59%)	59	1.48	0.118	1	0.731
**Cue A**	49(79.03%)	13(20.97%)	62	1.55	8.834	1	0.003*
**Cue B**	80(57.14%)	60(42.86%)	140	3.50	2.759	1	0.097
**Cue C**	5(27.78%)	13(72.22%)	18	0.45	9.708	1	0.002*
**Cue D**	40(64.52%)	22(35.48%)	62	1.55	0.140	1	0.709
**Cue E**	6(75%)	2(25%)	8	0.20	0.551	1	0.458
**Cue F**	3(60%)	2(40%)	5	0.13	0.013	1	0.910
**Total**	221	133	354	8.85	18.934	6	0.004*

* *p* < 0.01.

### Student immediate response to cues

Table 4 shows the multilevel logistic regression results for student immediate response (*reduce space*) to *Cue A* and *Cue C* considering other consultation and student level variables. In general, all models suggested that *Cue C*, *Turn Duration* and *Cue Time* had a positive association, whereas *Cue A* and *Expert Rating* had a negative association, with the probability of the occurrence of a *reduce space* response. Level 2 residual variance estimates and LR^1^ test results indicated that consultations across students were similar due to limited variability in Level 2 variables. The only exception is for *Expert Rating* (*OR* = 0.93, *p* < 0.05). The effect of *Cue Time Squared* (i.e. the nonlinear effect) on the log odds of *reduce space* was not significant (*OR* = 1.00, *p* > 0.05); nor was the effect of the *Patient Rating* (*OR* = 0.74, *p* > 0.05). Although LR^2^ test did not suggest that Model 5 was significantly better than Model 2 (χ^2^(1) = 0.04, *p* > 0.05), we considered Model 5 as the final model after gender effect was controlled. Therefore, using estimates in Model 5, occurrence of a *Cue C* stimulated *reduce space* (*OR* = 4.88, *p* = 0.006), while presence of a *Cue A* discouraged *reduce space* response (*OR* = 0.37, *p* = 0.005). This suggests that it was nearly five times more likely to observe a *reduce space* response following presence of a *Cue C*, compared to a *Cue A*. Please note the large confidence interval for *Cue C*, which suggested the estimate might be unreliable possibly due to a small frequency of *Cue C* observed. Furthermore, Model 5 estimates showed that the longer the patient speech turn where cues occurred (*OR* = 1.02, *p* = 0.016), and the closer the interaction was to the end of the consultation (*OR* = 1.01, *p* = 0.000), the higher the likelihood of students closing down on patient emotional cue disclosure. Practically speaking, every one second increase in the patient speech turn would result in a 2% increase in the likelihood of observing a *reduce space* response in students. Furthermore, every one second closer to the end of a consultation would induce a further 1% rise in the likelihood of the occurrence of the *reduce space* behaviour. Consequently, the expert examiner was less likely to rate the student with higher scores when a larger number of *reduce space* responses were observed (*OR* = 0.93, *p* = 0.045). 

**Table 4:** Multilevel logistic regression lag 1 sequence models for *reduce space* outcome.

	**Null**		**Model 1**			**Model 2**			**Model 3**			**Model 4**			**Model 5**	
**Fixed effects**		*OR*	95% CI	*P*	*OR*	95% CI	*P*	*OR*	95% CI	*P*	*OR*	95% CI	*P*	*OR*	95% CI	*P*
Cue A		0.35	0.18, 0.71	0.004	0.37	0.18, 0.74	0.005	0.36	0.18, 0.72	0.004	0.37	0.18, 0.74	0.005	0.37	0.18, 0.74	0.005
Cue C		5.02	1.62, 15.57	0.005	4.87	1.57, 15.07	0.006	4.89	1.58, 15.17	0.006	4.94	1.59, 15.36	0.006	4.88	1.58, 15.11	0.006
Turn Duration		1.03	1.01, 1.05	0.011	1.02	1.00, 1.04	0.015	1.02	1.00, 1.04	0.016	1.02	1.01,1.05	0.011	1.02	1.00, 1.04	0.016
Cue Time		1.01	1.00, 1.01	0.000	1.01	1.00, 1.01	0.000	1.01	1.00, 1.02	0.035	1.01	1.00, 1.01	0.000	1.01	1.00, 1.01	0.000
Cue Time Squared								1.00	0.99, 1.00	>0.05						
Expert Rating					0.93	0.87, 0.99	0.046	0.93	0.87, 0.99	0.049	0.95	0.87, 1.02	>0.05	0.93	0.86, 0.99	0.045
Patient Rating											0.74	0.39, 1.39	>0.05			
Gender (ref:female)														1.05	0.62, 1.78	>0.05
**Random effects (intercept)**																
Level2 variance	0.14		0.22			0.13			0.12			0.11			0.13	
(student)	(0.02,1.16)		(0.04,1.29)			(0.01,1.82)			(0.01,1.96)			(0.01,1.99)			(0.01,1.88)	
LR^1^ test	χ^2^(1)=1.26		χ^2^(1)=1.98			χ^2^(1)=1.75			χ^2^(1)=0.67			χ^2^(1)=0.64			χ^2^(1)=0.72	
	*p*=0.131		*p*=0.080			*p*=0.193			*p*=0.207			*p*=0.211			*p*=0.198	
Log likelihood	-233.69		-213.17			-211.28			-210.83			-210.84			-211.27	
			χ^2^(4)=41.04			χ^2^(1)=3.78			χ^2^(1)=0.91			χ^2^(1)=0.89			χ^2^(1)=0.04	
LR^2^ test			*p*=0.000			*p*=0.052			*p*=0.341			*p*=0.345			*p*=0.847	
			CMnull			*CM1			CM2			CM2			CM2	

* CM1= compared with Model 1

LR^1^ test = likelihood ratio test comparing the mixed effects logistic model to a standard logistic modelLR^2^ test = likelihood ratio test for model improvement

### Effects of cumulative number of cues on student response

Results suggested by the attrition models were generally consistent with those found in previous models using lag 1 sequential analysis approach. Result details are therefore not reported here, but are available upon request to the first author. Estimates in the final model indicated that a greater number of *Cue C* cumulatively disclosed by the patient resulted in an increased likelihood of students responding by *reduce space* (*OR* = 5.06, *p* = 0.005); conversely, the larger the cumulative number of *Cue A*, then students were less likely to provide a *reduce space* response (*OR* = 0.64, *p* = 0.027). The effect of *Expert Rating* became non-significant in all attrition models (*OR* = 0.93, *p*> 0.05).

## Discussion

This study describes how medical students manage emotional cues and concerns expressed by simulated patients in their OSCE consultations. Cues and concerns and student responses were defined by the VR-CoDES-CC and VR-CoDES-P respectively. The study tested the hypothesis that student response style (i.e. *reduce space* vs *provide space*) depended not only on specific type of cues, but also on the cumulative frequency of that cue expressed before the point when a particular response was offered. Analysis was conducted considering a nested structure of the data at both between and within consultation levels.

### Applicability of the VR-CoDES to the OSCE consultation context

Cohen’s Kappa for both inter- and intra-coder reliability was about 0.80, which suggested that the VR-CoDES can be reliably coded when applied to the OSCE consultation with medical students and simulated patients. Compared to other consultation contexts involving simulated patients [19], a relatively high occurrence (*n* = 8.85) of cues and concerns were identified per consultation, which was almost two cues and concerns per minute, given the average duration of an OSCE consultation was about five minutes. This might be partly explained by the highly emotion-provoking nature of the IBS scenario used for that session. Furthermore, virtually all cue types (with the exception of *Cue G*) and concerns, including *Cue E* (patient elicited repetition) and *Cue F* (nonverbal cues), were identified with the VR-CoDES-CC, suggesting that the VR-CoDES is capable of differentiating cues in this context. On the other hand, it was not surprising to experience difficulties in discriminating a *Cue A* from a *concern* on a few occasions. Regarding response coding, distinguishing between *explicit* and *non-explicit* dimension proved challenging for certain codes, the majority of other codes were relatively easily identified and agreed upon. 

### Do medical students respond differently (provide space vs reduce space) to different cue types?

The findings suggested that students responded by providing space to emotional cues expressed in vague and unspecified words that were verbally close to stating a concern (*Cue A*). This can be interpreted as being consistent with how young psychiatrists responded by passive listening to all concerns expressed by simulated patients [19]. ‘Hmm’, ‘Yeah’ and ‘echoing’, some key features coded as patient-centred techniques of passive listening in the VR-PICS [19] were the main aspects considered as a *provide space* response to cues commonly coded as *Back Channel* in the VR-CoDES [24]. Apart from ‘yes, right, hmm’ type of non-explicit *provide space* response, other typical examples included ‘Could you tell me more about it?’ following a patient’s *concern* (e.g., ‘I am worried about this episode.’) or a *Cue A* (e.g., I am very stressed at work at the moment.’). When cues were expressed emphasizing physiological or cognitive correlates (*Cue C*), students were more likely to close down on the patient emotional disclosure. This might be explained by medical students being inexperienced in dealing with emotions that were seemingly unrelated to the topics (e.g., complaining about not being able to concentrate on work) especially when working in a pressurised situation. Students often responded to this type of complaint (coded as *Cue C*) with ‘Do you have any allergy?’ or ‘Could I ask you if you are otherwise fit and well?’. These *reduce space* responses indicated that students felt pressurised possibly due to OSCE time constraints, which was confirmed by our data that medical students were more likely to *reduce space* to cues when the consultation was nearer its end. There were no systematic effects found with concerns and for the other cues to help predict responses (*provide* or *reduce space*). We believe that this is the first report that has investigated the differential effects of different cues on responses. 

### Do cumulative frequencies of cues have an effect on the occurrence of the reduce space response?

This hypothesis was supported as similar results were found with ‘attrition’ models investigating effects on cumulative frequency of cues on responses. It would appear that the cumulative frequency of cues does not lend improved explanation of the *reduce space* response of medical students. This may support the view that students are not noticing repeated cue types in a consultation and responding differently to such repetition. It might also be possible that the same ***cuetype*** (e.g., ‘I am very stressed at work’; ‘I am fed up with it’; and ‘I am embarrassed about this’ are all coded as *Cue A*) were less sensitive to detect response differences, compared to examining the effect of cumulative frequency of the ***samecue*** (e.g., ‘I am very stressed at work at the moment’ only.) Alternatively, the data set may have been limited in size and underpowered to isolate these additional effects. To discriminate a parsimonious model from other models, further investigation is needed, with a stronger theoretical basis and more rigorous design, to enhance our understanding of the communication process [36].

### Is response type (*provide space vs reduce space*) related to the OSCE outcome rated by an expert examiner and the patient?

Our models indicated that the probability of the *reduce space* response is enhanced by the addition of the knowledge of the expert examiner. This effect can be either interpreted as experts identifying *reduce space* as being generally less competent; or specifically as being poorer at acknowledging patient concerns and responding in an empathic way. Patients’ perceptions of whether they were responded appropriately were not found to be significantly related to students’ *reduce space* behaviour. This might be due to the scoring system (i.e. from 0 to 2) not being sensitive enough to capture the response difference or to a small sample size being unable to detect a small effect. 

### Strengths and limitations of the study

A small sample size of student participants has limited our ability to generalize the findings to a wider population. In particular, when the heterogeneous sample may increase external validity, it was nevertheless difficult to compare the response differences between students with varied level of previous communication skills. Future studies can explore, with sufficient and more balanced sample sizes, how students’ past OSCE performance might influence the way they respond to cues and concerns. In addition to the sample limitation, limited variability in Level 2 variables (e.g. patient rating scores) has restricted the evaluation of random effects and interaction effects among significant variables in the final models. A final limitation is related to the nature of the OSCE scenario (IBS). It is uncertain whether the attrition model serves to promote understanding of the communication processes when applied to different scenarios. It will be interesting to explore further whether utilisation of the lag independent relationship approach is dependent on the nature of the consultation content. To improve our understanding of the usefulness of the attrition model in healthcare communication process, future researchers are also encouraged to withstand statistical challenges to distinguish the effect of cumulative frequency of the same cue type versus the same cue. 

To our knowledge only two studies [19,21] have so far investigated provider response to patient emotionally charged expressions using a multilevel analysis approach. While the other two studies were specifically applied to psychiatric consultations, this is the first to investigate medical students’ responses to emotional cues and concerns in OSCE consultation contexts distinguishing effects acting at different levels. In response to the current promotion of a patient-centred communication approach, the findings have implications for communication skills training and clinical practice [37,38]. 

A key strength of the study is that the statistical flexibility in the approach we adopted enabled us to test hypotheses derived from a developing theoretical basis. This is in contrast to a largely explorative nature of the studies conducted in the past [22]. Complemented by the attrition model, communication processes can now be investigated from both immediate and cumulative influences. Researchers have been encouraged to find appropriate methods to match their hypotheses rather than constraining theoretical reasoning to fit familiar methods [22]. Our study is one attempt to test alternative models with a relatively flexible and powerful set of analytical techniques. 

Our attempt to link student response behaviour with examiner and patient rating can be also seen as a merit of the study seeking to associate components of healthcare communication to healthcare outcomes, which has been attracting an increasing attention in clinical communication research [39–41]. A deliberate design feature of this study was to incorporate accurate time stamps for all the utterances that possessed a cue or concern. This enabled the effects of time when the cue or concern was expressed and thereby controlled for the increased likelihood of students’ *reduce space* response when nearing the end of the consultation. A strong linear effect was confirmed showing that students were more likely to close space nearer the end of the consultation. However the prediction of the *reduce space* variance was not improved when introducing a squared term. The student would appear then not to close down systematically, at a much greater probability, when the five minute duration (approximately) was approaching.

## Conclusions

The VR-CoDES showed to be reliable when applied to the OSCE consultation contexts. Medical students offered responses that differed to cue types expressed by simulated patients. Students appeared to *provide space* to cues when expressed in vague and unspecific words (*Cue A* defined in the VR-CoDES) that is verbally close to stating a concern; and *reduce space* to cues emphasizing physiological or cognitive correlates (*Cue C* defined in the VR-CoDES). In addition, students were more likely to offer *reduce space* response nearer the end of the consultation, and when the duration of a patient speech turn got larger. Students’ *reduce space* response was also predicted by the cumulative frequency of cues. 

Studying how medical students handle negative emotions has significant implications for training programme development focusing on student emotion recognition skills and patient-centred communication approach. Hence, studies of this type will also have important implications for clinical practice. In addition, the statistical approach adopted in this study, combining lag 1 sequential technique and attrition models, will encourage researchers to search for appropriate analytical techniques to test theoretical propositions in healthcare communication research. 
